# Cytoprotective Effect of Short-Term Pretreatment with Proanthocyanidin on Human Gingival Fibroblasts Exposed to Harsh Environmental Conditions

**DOI:** 10.1371/journal.pone.0113403

**Published:** 2014-11-18

**Authors:** Michiko Kurauchi, Yoshimi Niwano, Midori Shirato, Taro Kanno, Keisuke Nakamura, Hiroshi Egusa, Keiichi Sasaki

**Affiliations:** Tohoku University Graduate School of Dentistry, Aoba-ku, Sendai, Japan; University of Dhaka, Bangladesh

## Abstract

Our previous study showed that exposing mouse fibroblasts to proanthocyanidin (PA) for only 1 min accelerated cell proliferation in a concentration-dependent manner. In this study, exposing human gingival fibroblasts (HGFs) to PA for 1 min similarly accelerated the proliferative response of the cells. Besides the accelerated proliferative response, PA showed a cytoprotective effect on HGFs exposed to harsh environmental conditions; short-term exposure of HGFs in the mitotic phase to pure water or physiological saline resulted in a lower recovery of viable cells. Pretreatment and concomitant treatment with PA improved the low recovery of cells exposed to pure water or physiological saline. In addition, HGFs exposed to PA for 1 min proliferated well even after being cultured in serum-free medium. In 100% confluent HGFs, being cultured in serum-free medium resulted in a high intracellular reactive oxygen species (ROS) level, but pretreatment with PA prevented the cells from increasing intracellular ROS. Thus, the results suggest that a short-term PA treatment exerts cytoprotective effects on HGFs exposed to harsh environmental conditions by improving the intracellular oxidative stress response.

## Introduction

Proanthocyanidin (PA) is a group of polyphenolic compounds that naturally occur in fruits, vegetables, nuts, seeds, and flowers [1]; PA is a polymer of flavan-3-ols, such as (+)-catechin, (−)-epicatechin, and (−)-epicatechin gallate, with an average degree of polymerization between 2 and 17 [2], [3]. Our previous study revealed that exposing mouse fibroblasts to PA for only 1 min accelerated cell proliferation in a concentration-dependent manner [4]. In addition, intracellular reactive oxygen species (ROS) formation induced by hydrogen peroxide was significantly inhibited in the cells that were pretreated with PA for 1 min, suggesting that the PA that was incorporated into the cells within 1 min sufficiently exerted cytoprotective effects on the cells suffering from oxidative stress. It was reported that the reduced cell viability and oxidative stress in HepG cells induced by tert-butyl hydroperoxide were protected by treatment with PA for 6 h [5]. In addition, recent studies demonstrated the cytoprotective effects of polyphenols. For instance, ellagic acid ameliorated the cytotoxic effect of paraquat (1,1′-dimethyl-4,4′-bipyridinium dichloride) on human alveolar A549 cells via its antioxidant action [6]; pretreatment with resveratrol up-regulated the expression of methionine sulfoxide reductase A in human neuroblastoma SH-SY5Y cells, resulting in the enhanced resistance of the cells to neurotoxins [7]. Epigallocatechin-3-gallate showed a cytoprotective effect on mycotoxin-induced cytotoxicity in human colon adenocarcinoma cell line HT29 cells through anti-oxidative and anti-inflammatory mechanisms [8]; brain-accessible polyphenols might have a protective effect on neurodegeneration through the sirtuin pathway [9]. Lemon grass (*Cymbopogon citratus* Stapf) polyphenols protected human umbilical vein endothelial cells from oxidative damage induced by high glucose, hydrogen peroxide, and oxidized low-density lipoprotein [10]. A major difference between these studies and our study was the PA treatment time. We further examined the cytoprotective effect of short-term PA treatments on human gingival fibroblasts (HGFs) exposed to stressful conditions to search for a novel protective and therapeutic agent against oral injury.

## Materials and Methods

### Test substance, cell culture, and cell proliferation assay

PA (Leucoselect) was purchased from Indena (Milano, Italy). HGFs were purchased from Primary Cell Co., Ltd. (Sapporo, Japan). Dulbecco's modified Eagle's medium (DMEM, Thermo Fisher Scientific, Waltham, MA, USA) containing 10% fetal bovine serum (FBS, Thermo Fisher Scientific), 100 U/mL penicillin (Wako Pure Chemicals Industries Ltd, Osaka, Japan), and 0.1 mg/mL streptomycin (Wako Pure Chemicals Industries Ltd) was used as a medium for cell culture. An aliquot (100 µL) of the cell suspension (2×10^4^ cells/mL) was placed in each well of a 96-well culture plate. The plates were incubated at 37°C in humidified 5% CO_2_ for 24–28 h for sub-confluence (10–20%) or 4–5 days for 100% confluence. After treatment, the cells were incubated for the designated time period to determine the cell viability by the methyl thiazolyl tetrazolium (MTT) assay [11], [12] or the neutral red (NR, Wako Pure Chemicals Industries Ltd) uptake assay [13], [14]. In the MTT assay, an insoluble formazan converted from MTT was colorimetrically determined at 595 nm by a microplate reader (FilterMax F5; Molecular Devices, Sunnyvale, CA, USA). The MTT assay was performed using a kit (TACS MTT Cell Proliferation Assay; Trevigen Inc., Gaithersburg, MD, USA). In the NR assay, the medium was replaced with medium containing 150 µg/mL NR and incubated for 3 h. Then, NR was extracted with 50% ethanol containing 1% acetic acid for colorimetric determination at 540 nm using the microplate reader (FilterMax F5).

### Cell proliferation after a 1-min PA pretreatment

After cells reached the sub-confluent condition, the medium was replaced with sterile physiological saline or saline containing different concentrations of PA. After 1 min, the cells were washed and cultured in fresh medium for 24 h to determine the cell viability by the MTT assay.

### Cell proliferation after exposure to pure water or physiological saline

After cells reached the sub-confluent condition, the medium was replaced with pure water or pure water containing 1 mg/mL PA. After 1, 4, and 16 min at room temperature, the cells were washed and cultured in fresh medium for 24 h to determine the cell viability by the MTT assay. Similarly, when the cells reached the sub-confluent condition, the medium was replaced with physiological saline or saline containing 1 mg/mL PA. After 1, 2, and 3 h at room temperature, the cells were washed and cultured in fresh medium for 22–24 h to determine the cell viability by the MTT assay. The effect of pretreatment with PA was also examined. After cells reached the sub-confluent condition, the cells were treated with sterile physiological saline or saline containing 1 mg/mL PA for 1 min. After washing, the cells were exposed to pure water for 1 min or physiological saline for 1 h. Then, the cells were washed and cultured in fresh medium for 24 h to determine the cell viability by the MTT assay.

### Proliferation and intracellular ROS in HGFs cultured in serum-free medium

After cells reached the sub-confluent condition, the medium was replaced with physiological saline or saline containing 1 mg/mL PA. After 1 min, the cells were washed and cultured in serum-free medium (*i.e.*, DMEM without serum supplementation) for 24, 48, and 72 h to determine the cell viability by the MTT assay. In an experiment where intracellular ROS was determined, after cells reached 100% confluence, the medium was replaced with physiological saline or saline containing 1 mg/mL PA. After 1 min, the cells were washed and cultured in serum-free medium for 24 h to determine the cell viability and intracellular ROS. The cell viability was determined by the NR assay, and intracellular ROS levels were determined using a kit (Oxiselect Intracellular ROS Assay Kit; Cell Biolabs, Inc., San Diego, CA, USA). In this assay, a cell-permeable probe, 2′,7′-dichlorodihydrofluorescin diacetate (DCFH-DA) diffuses into cells and is deacetylated to a nonfluorescent product, 2′,7′-dichlorodihydrofluorescin (DCFH), by cellular esterases; in the presence of cytosolic ROS, DCFH is oxidized to a highly fluorescent molecule, 2′,7′-dichlorodihydrofluorescein (DCF) [15].

### Statistical analysis

The statistical significance (p<0.05) of viable cells as expressed as the percentage of control, and the amount of intracellular ROS were assessed by one-way analysis of variance followed by the Dunnett's multiple comparison test or the Tukey–Kramer honestly significant difference comparison test.

## Results and Discussion

**Figure 1:**
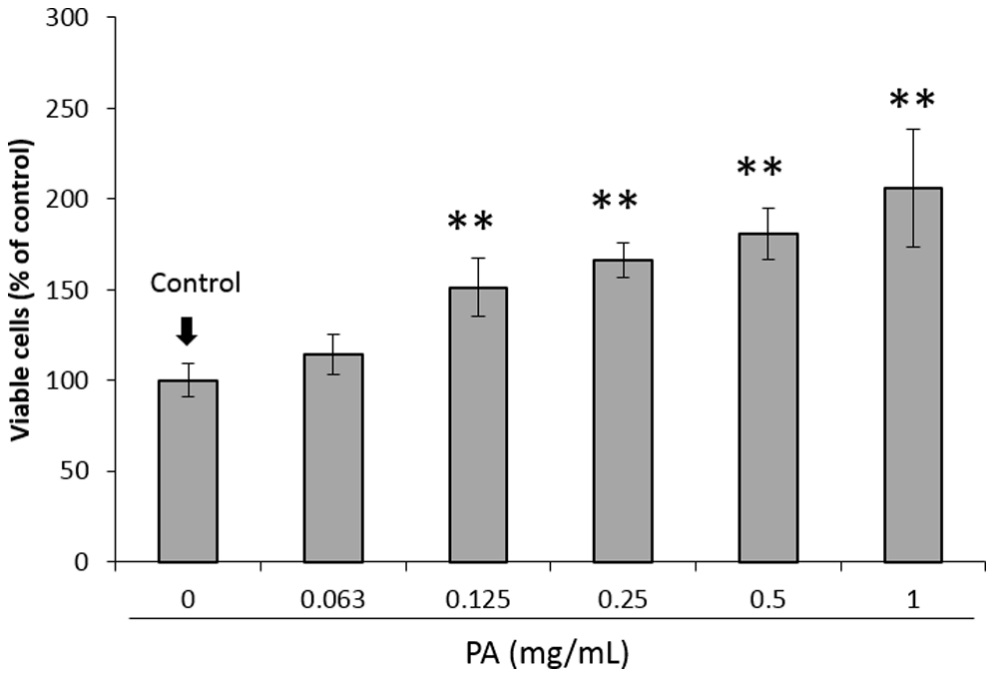
Effect of pretreatment with different concentrations of proanthocyanidin (PA) for 1 min on the proliferative response of human gingival fibroblasts (HGFs). Each value represents the mean ± standard deviation (n = 4). Significant differences from the control group are shown as ** p<0.01 (Dunnett's multiple comparison test).

Figure 1 shows the proliferative response of the subconfluent HGFs 24 h after the 1-min PA treatment. The PA pretreatment significantly accelerated the proliferative response of the cells in a concentration-dependent manner. Besides the accelerated proliferative response, PA showed a cytoprotective effect on cells that were exposed to harsh environmental conditions.

**Figure 2:**
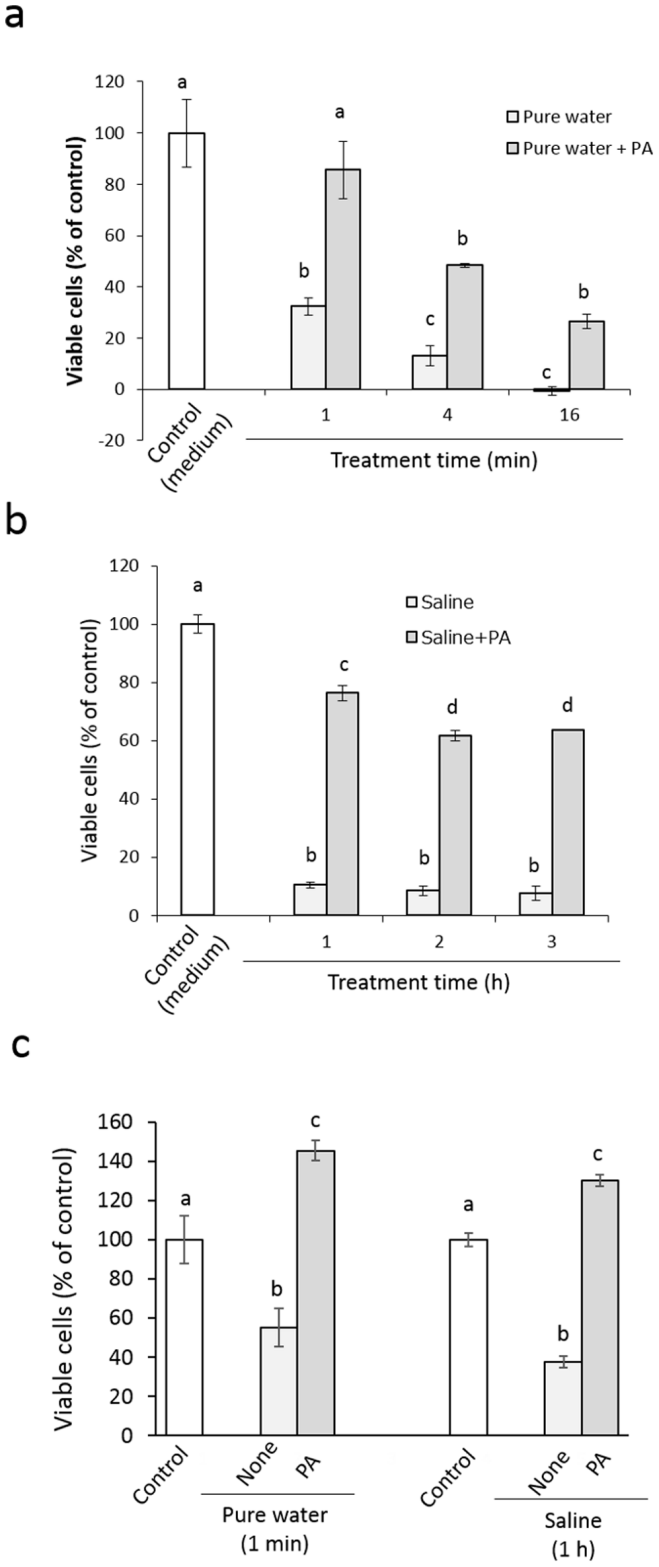
Effect of concomitant treatment with 1 mg/mL PA on the proliferative response of HGFs exposed to pure water for 1–16 min (a) or physiological saline for 1–3 h (b), and the effect of a 1-min pretreatment with PA on the proliferative response of HGFs exposed to pure water for 1 min or physiological saline for 1 h (c). Each value represents the mean ± standard deviation (n = 4). Significant differences (p<0.05, Tukey–Kramer multiple comparison test) within each group are denoted by different letters (*i.e.*, bars with the same letter are not significantly different).

Figure 2 shows the viability of HGFs after short-term exposure to pure water or physiological saline. When subconfluent cells were exposed to pure water for 1–16 min, the cell viability 24 h after the exposure was significantly lowered in an exposure-time-dependent manner compared with that in the untreated control group (Fig. 2a). This reduced cell viability was improved by PA. That is, once 1 mg/mL PA was present concomitantly with pure water, the recovered cell viability after 24 h was significantly higher than that of the corresponding pure water-exposed group. Similarly, when subconfluent cells were exposed to physiological saline for 1–3 h, the cell viability 24 h after the exposure was significantly lowered by more than 90% of that of the untreated control group (Fig. 2b). This lowered cell viability was also improved by PA. As with the pure water, once 1 mg/mL PA was present concomitantly with the saline, the recovered cell viability after 24 h was significantly higher than that in the corresponding saline-exposed group. Figure 2c shows the effect of 1-min PA pretreatment on the viability of cells 24 h after exposure to pure water for 1 min or physiological saline for 1 h. Similar to the results in the experiments of concomitant treatment with PA, a 1-min pretreatment with PA resulted in significantly higher cell viability than that in the pure water- or saline-exposed group.

**Figure 3:**
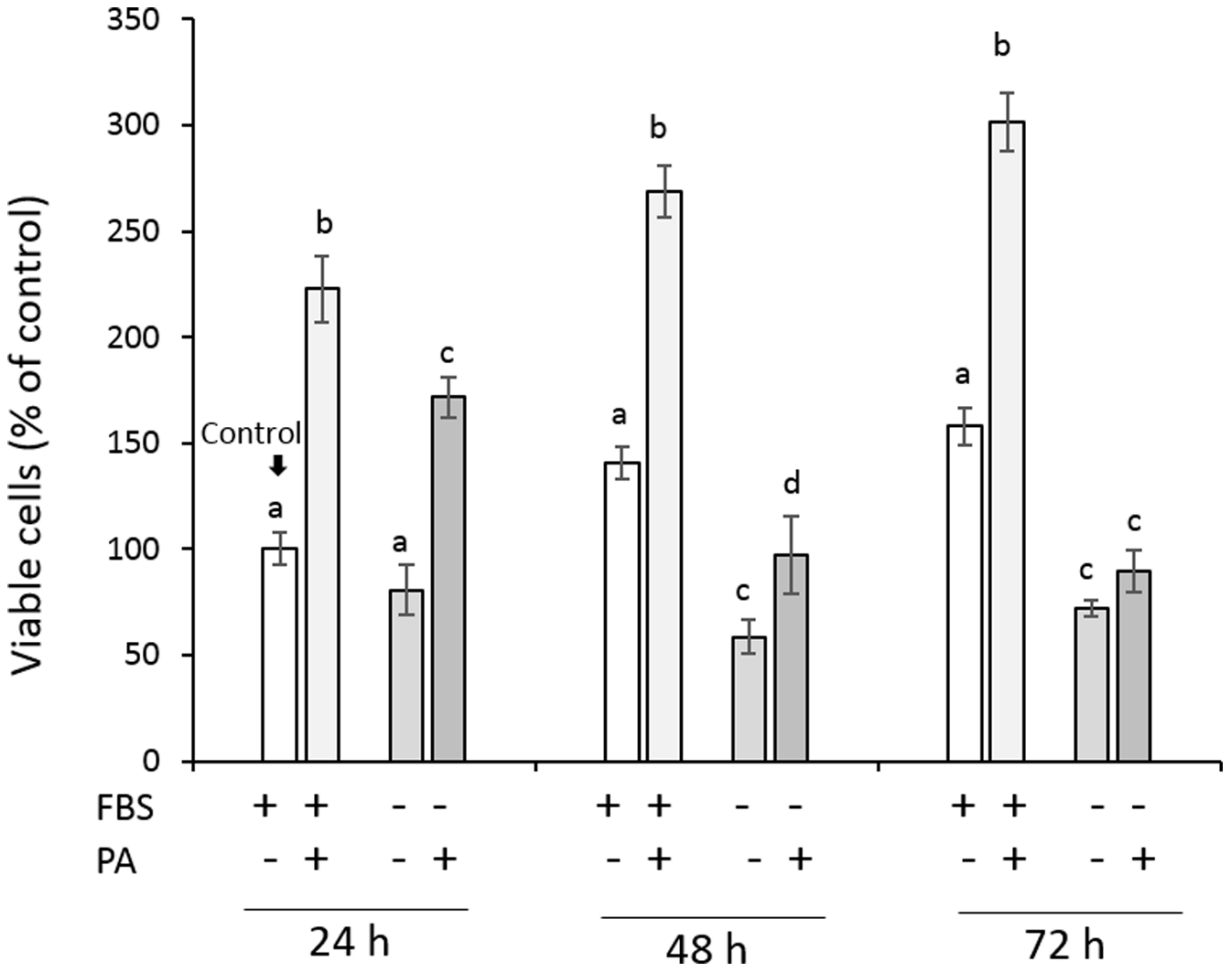
Effect of a 1-min pretreatment with PA (1 mg/mL) on the proliferative response of HGFs cultured in serum-free medium for 24–72 h. Each value represents the mean ± standard deviation (n = 4). Significant differences (p<0.05, Tukey–Kramer multiple comparison test) within each group at each time point are denoted by different letters (*i.e.*, bars with the same letter are not significantly different).

**Figure 4:**
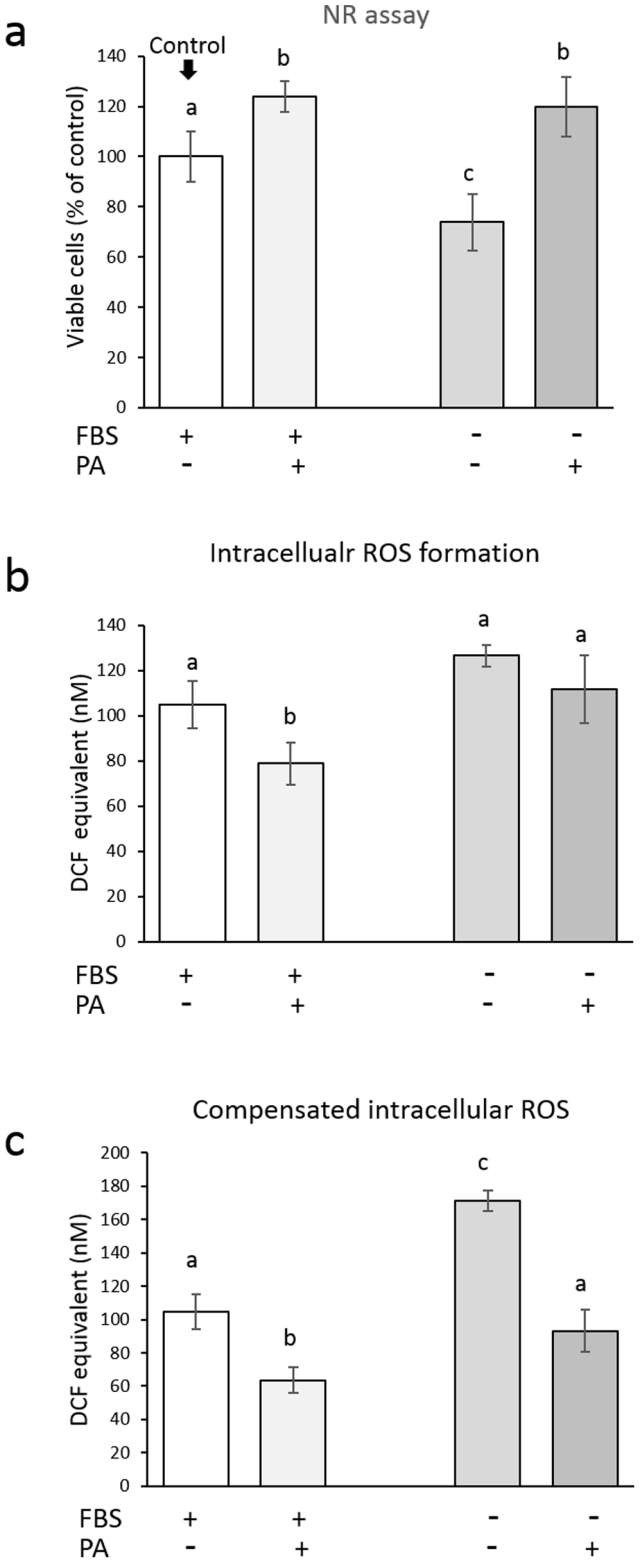
Effect of a 1-min pretreatment of 100% confluent HGFs with PA (1 mg/mL) on the cell viability (a) of, the amount of intracellular ROS per well (b) in, and the compensated amount of intracellular ROS per viable cell (c) in HGFs cultured in serum-free medium for 24 h. Since DCF was used as a standard, intracellular ROS level is expressed as DCF equivalent (nM). Each value represents the mean ± standard deviation (n = 4). Significant differences (p<0.05, Tukey–Kramer multiple comparison test) within each group are denoted by different letters (*i.e.*, bars with the same letter are not significantly different).

Figure 3 shows the viability of HGFs cultured in serum-free medium. The cell viability of the control group that was cultured in medium with 10% FBS increased with time, and pretreatment with 1 mg/mL PA significantly accelerated the proliferative response of the cells. When subconfluent cells were cultured in serum-free medium, the recovered cell viability gradually lessened with time; after 48 and 72 h, the cell viability was significantly lower than that of the corresponding control group. This reduced cell viability was improved when the cells were pretreated with 1 mg/mL PA; after 24 and 48 h, the cell viability was significantly higher than that in the corresponding serum-deprived group. After 72 h, no significant difference was found between the PA-pretreated group and the serum-deprived group. Because several reports demonstrated that serum deprivation induces apoptosis in relation to increased intracellular ROS [16]–[19], the effects of PA pretreatment on the intracellular ROS in HGFs cultured in serum-free medium were examined. Simultaneously, the cell viability was also determined to compare the amount of ROS per well and amount of ROS per viable cell. Unlike the observations of the subconfluent cells, the formazan formation by the reduction of MTT in the 100% confluent cells might be affected by the incorporation of PA into the cells. In addition, if serum deprivation induces mitochondrial dysfunction, it might result in increased leakage of electron with subsequent reduction of molecular oxygen to form ROS. Thus, the MTT assay might not reflect living cells proportionally, as a result, the NR assay, but not the MTT assay, was applied to determine the cell viability in this experiment. Figure 4 shows the intracellular ROS level relative to the cell viability 24 h after the treatment of 100% confluent cells with 1 mg/mL PA for 1 min. When the 100% confluent cells were cultured in serum-free medium for 24 h, the cell viability was significantly lower than that of the control group cultured in medium with 10% FBS (Fig. 4a), although no significant difference was found in the intracellular ROS level per well between the two groups (Fig. 4b). Accordingly, the amount of intracellular ROS per viable cell, as determined by the NR assay, was significantly increased by serum deprivation (Fig. 4c) as reported previously [16]–[19]. This reduction in cell viability was prevented by the 1-min PA pretreatment. That is, the viability of the cells pretreated with 1 mg/mL PA for 1 min was significantly higher than that of the serum-deprived group. Nonetheless, the intracellular ROS levels per well were nearly similar, so that that amount of compensated intracellular ROS became much lower in the PA-pretreated group than in the serum-deprivation group. In addition, even when the cells were cultured in medium with 10% FBS, the intracellular ROS level in the PA-pretreated cells was significantly lower than that in the control group.

Regarding the localization of PA, attachment of PA to the cell membrane is one of the possibilities for its ability to protect cells. Thus, we examined this point in the previous study using mouse fibroblasts as in the following way [4]. If incorporated PA for such a short time as 1 min accelerates the proliferation response, a bioassay was conducted by utilizing antioxidant potential of PA [20]. That is, oxidative stress was induced to the cells pretreated with PA. As a result, intracellular oxidative stress was significantly suppressed in the cells pretreated with PA for 1 min, suggesting that incorporated PA into the cells exerted antioxidant effect on the oxidative stress. Mass spectrometry analysis also revealed that PA could be incorporated into the cells within 1 min [4]. Thus, in this study, it is presumed that PA incorporated into HGFs exerted cytoprotective effects. One mechanism by which incorporated PA exerts its effects is a direct antioxidant effect on the oxidative stress caused, for instance, by accumulated ROS, as shown in the serum deprivation experiment, and another mechanism is an indirect effect through the up-regulated gene expression related to the antioxidant defense mechanism. Indeed, it was reported that the Nrf2/antioxidant defense pathway, which is a crucial cellular defense mechanism that protects cells from oxidative stress [21], was activated through the up-regulation of sirtuin 1 by resveratrol, a naturally occurring polyphenol found in red wine [22]. We will further examine if the same mechanism acts in not only the cytoprotective effect of PA on HGFs exposed to pure water (*i.e.*, osmotic stress) and physiological saline (*i.e.*, starvation stress) but also the accelerating effect of PA pretreatment on the proliferative response of the HGFs. Regardless of the cytoprotective mechanism of PA, PA prevents cells from damage by detrimental agents such as ROS released from immune cells (*e.g.*, neutrophils and macrophages) upon microbial infection [22]; as such, PA could be a novel therapeutic agent for oral injury in terms of its cytoprotective effect.
